# Supplementary material for: The Systems Biology Research Tool: evolvable open-source software
Source: BMC Syst Biol. 2008 Jun 29;2:55. doi: 10.1186/1752-0509-2-55 (PMC2446383; doi:10.1186/1752-0509-2-55)
Supplement: Additional file 1 — SBRT Archive. An archive of the current version of the Systems Biology Research Tool. [file 1752-0509-2-55-S1.zip › sbrt-1.4.0/doc/developers_guide/api/sbrt/shell/mng/fba/FbaManager.html]

FbaManager


|  |  |  |
| --- | --- | --- |
| **Overview** \| **Package** \| **Class** \| **Use** \| **Tree** \| **Deprecated** \| **Index** \| **Help** | |  |
| **PREV CLASS**   **NEXT CLASS** | **FRAMES**    **NO FRAMES**     **All Classes** |  |
| SUMMARY: NESTED \| FIELD \| CONSTR \| METHOD | DETAIL: FIELD \| CONSTR \| METHOD |  |


---


## sbrt.shell.mng.fba Class FbaManager

```
java.lang.Object
  sbrt.shell.mng.AbstractProcessManager
      sbrt.shell.mng.fba.FbaManager
```

**All Implemented Interfaces:**: KernelProcessManager<java.lang.String,java.lang.String>, ProcessManager<java.lang.String,java.lang.String>

**Direct Known Subclasses:**: CatalystDeletionManager, CvCatalystDeletionManager, CvFbaOptManager, CvMsFluxIntervalManager, CvObjectiveFunctionManager, CvRevRxnFluxIntervalManager, CvRxnDeletionManager, CvSimpleFluxIntervalManager, ExtremeCurrentManager, FbaOptManager, FbaSolverManager, FluxActivityManager, FluxCapManager, FluxPlasticityManager, InitialPointManager, IntervalVariationManager, MsFluxIntervalManager, MsFluxomeReducerManager, NetworkInfoManager, ObjectiveFunctionManager, RandomConstraintsManager, RandomEnvManager, RandomFluxVectorManager, RandomObjectiveManager, ReverseRxnFluxIntervalManager, RxnDeletionManager, SimpleFluxIntervalManager, SlpCycleIdManager, StoichVariationManager, SvCdHarFluxVectorManager, SvInitialPointManager, WwFluxomeReducerManager

---

``` public abstract class FbaManager extends AbstractProcessManager implements KernelProcessManager<java.lang.String,java.lang.String> ```

This abstract class provides a foundation for Flux Balance Analysis
managers. It contains many of the keywords required by FBA managers, as well
as a set of utility functions.

**Author:**
:   This class was written and documented by Jeremiah Wright while
    in the Wagner lab.

---

| **Field Summary** | |
| --- | --- |
| `static java.lang.String` | `CONSTRAINT_TOLERANCE`             The keyword used to denote a constraint tolerance. |
| `static double` | `DEFAULT_CONSTRAINT_TOLERANCE`             The default constraint tolerance. |
| `static java.lang.String` | `FLUX_VECTOR_FILE`             The keyword used to denote a flux vector input file. |
| `static java.lang.String` | `FLUX_VECTOR_FILE_NAME`             The keyword used to denote the name of a flux vector output file. |
| `static UnmodifiableSet<java.lang.String>` | `optionalKeywords`             The set of keywords understood, but not required, by instances of this class. |
| `static UnmodifiableSet<java.lang.String>` | `requiredKeywords`             The set of keywords required to be present in the `ManagerInput` objects supplied to instances of this class. |
| `static java.lang.String` | `RXN_CATALYST_FILE`             The keyword used to denote the name of a file containing reaction-catalyst associations. |
| `static java.lang.String` | `RXN_FILE`             The keyword used to denote an FBA reaction file. |
| `static java.lang.String` | `SYSTEM_SOLUTION_FILE`             The keyword used to denote a file containing a solution to the system of linear equations formed by a fluxome. |

**Fields inherited from class sbrt.shell.mng.AbstractProcessManager**

`ALGORITHM, COMPLETED, DATA_HEADERS, DEV_NULL, ELAPSED_TIME, FILE_FORMAT, INPUT_FILE, INPUT_FILE_FORMAT, INPUT_FILE_NAME_FILE, ITERATIONS, OUTPUT_FILE_FORMAT, OUTPUT_FILE_NAME, OUTPUT_FILE_NAME_FILE, PERCENT, PERCENT_COMPLETED, SEED, ZERO_CUTOFF`


**Constructor Summary**

`FbaManager()`


| **Method Summary** | |
| --- | --- |
| `static java.util.Map<java.lang.String,Or<And<java.lang.String>>>` | `getCatalysts(Fluxome fluxome, java.lang.String fileName)`             Returns the reaction-catalyst associations contained in the file with the provided name. |
| `static java.util.Map<java.lang.String,Or<And<java.lang.String>>>` | `getCatalysts(Fluxome fluxome, java.lang.String fileName, java.io.PrintWriter out)`             Returns the reaction-catalyst associations contained in the file with the provided name. |
| `static double` | `getConstraintTolerance(ManagerInput<java.lang.String,java.lang.String> input)`             Parses and returns the value for `CONSTRAINT_TOLERANCE` in the provided manager input. |
| `static double` | `getConstraintTolerance(ManagerInput<java.lang.String,java.lang.String> input, java.io.PrintWriter out)`             Parses and returns the value for `CONSTRAINT_TOLERANCE` in the provided manager input. |
| `static IrrevRxnFluxome` | `getFluxome(ManagerInput<java.lang.String,java.lang.String> input, java.io.PrintWriter out)`             Opens the file denoted by `RXN_FILE` in the provided manager input and returns the fluxome it contains. |
| `static IrrevRxnFluxome` | `getFluxome(java.lang.String fileName)`             Returns the fluxome contained in the file with the provided name. |
| `static IrrevRxnFluxome` | `getFluxome(java.lang.String fileName, java.io.PrintWriter out)`             Returns the fluxome contained in the file with the provided name. |
| `static IrrevRxnFluxome` | `getFluxome(java.lang.String rxnFileName, java.lang.String catalystFileName)`             Returns the fluxome contained in the files with the provided names. |
| `static IrrevRxnFluxome` | `getFluxome(java.lang.String rxnFileName, java.lang.String catalystFileName, java.io.PrintWriter out)`             Returns the fluxome contained in the files with the provided names. |
| `static LinearSystemSolution<java.lang.String>` | `getFluxomeSolution(ManagerInput<java.lang.String,java.lang.String> input, java.io.PrintWriter out, Fluxome fluxome)`             Opens the file denoted by `SYSTEM_SOLUTION_FILE` in the provided manager input and returns the linear system solution it contains. |
| `static MapOutputFile<java.lang.String,java.lang.Double>` | `getMultiFluxVectorOutputFile(ManagerInput<java.lang.String,java.lang.String> input, java.io.PrintWriter out, Fluxome fluxome)`             Returns an empty output file that is suitable for storing multiple flux vectors. |
| `static MapOutputFile<java.lang.String,java.lang.Double>` | `getMultiFluxVectorOutputFile(java.lang.String fileName, FileFormat fileFormat, java.util.Set<java.lang.String> rxnNameHeaders, Fluxome fluxome)`             Returns an empty output file that is suitable for storing multiple flux vectors. |
| `static RxnNameExprFormat<LinearComb<java.lang.String>,java.lang.String>` | `getRxnNameExprFormat(Fluxome f)`             Returns the default reaction name expression format. |
| `static java.util.Set<java.lang.String>` | `getRxnNameHeaders(ManagerInput<java.lang.String,java.lang.String> input, Fluxome fluxome)`             Parses and returns the value for `AbstractProcessManager.DATA_HEADERS` in the provided manager input. |
| `static java.util.LinkedHashSet<java.lang.String>` | `getRxnNameHeaders(java.lang.String dataHeaders, Fluxome fluxome)`             Parses and returns the set of reaction names contained in the provided string. |
| `static MapOutputFile<java.lang.String,java.lang.Double>` | `getSingleFluxVectorOutputFile(ManagerInput<java.lang.String,java.lang.String> input, java.io.PrintWriter out, Fluxome fluxome)`             Returns an empty output file that is suitable for storing a single flux vector. |
| `static MapOutputFile<java.lang.String,java.lang.Double>` | `getSingleFluxVectorOutputFile(java.lang.String fileName, FileFormat fileFormat, java.util.Set<java.lang.String> rxnNameHeaders, Fluxome fluxome)`             Returns an empty output file that is suitable for storing a single flux vector. |
| `static void` | `printFluxomeInfo(Fluxome fluxome, java.io.PrintWriter out)`             Prints basic information about the provided fluxome. |
| `static void` | `writeFluxome(IrrevRxnFluxome fluxome, java.lang.String fileName)`             Writes the provided fluxome to a file with the provided name. |
| `static void` | `writeFluxome(IrrevRxnFluxome fluxome, java.lang.String fileName, java.io.PrintWriter out)`             Writes the provided fluxome to a file with the provided name. |

**Methods inherited from class sbrt.shell.mng.AbstractProcessManager**

`check, closeFile, closeRm, execute, getFileFormat, getFileFormat, getFileNameSource, getIterations, getSeed, getTextFileFormat, getTextFileFormat, getZeroCutoff, getZeroCutoff, parseInt, parseLong, printProgress, printProgress, printProgress, runProcess, setInput`

**Methods inherited from class java.lang.Object**

`clone, equals, finalize, getClass, hashCode, notify, notifyAll, toString, wait, wait, wait`

**Methods inherited from interface sbrt.shell.mng.KernelProcessManager**

`getProcess`

**Methods inherited from interface sbrt.shell.mng.ProcessManager**

`check, execute, execute, getOptionalKeys, getRequiredKeys, setInput, setInput`

**Field Detail**

### RXN\_FILE

```
public static final java.lang.String RXN_FILE
```

:   The keyword used to denote an FBA reaction file.

    **See Also:**: Constant Field Values

---


### RXN\_CATALYST\_FILE

```
public static final java.lang.String RXN_CATALYST_FILE
```

:   The keyword used to denote the name of a file containing reaction-catalyst
    associations.

    **See Also:**: Constant Field Values

---


### FLUX\_VECTOR\_FILE

```
public static final java.lang.String FLUX_VECTOR_FILE
```

:   The keyword used to denote a flux vector input file.

    **See Also:**: Constant Field Values

---


### FLUX\_VECTOR\_FILE\_NAME

```
public static final java.lang.String FLUX_VECTOR_FILE_NAME
```

:   The keyword used to denote the name of a flux vector output file.

    **See Also:**: Constant Field Values

---


### SYSTEM\_SOLUTION\_FILE

```
public static final java.lang.String SYSTEM_SOLUTION_FILE
```

:   The keyword used to denote a file containing a solution to the system
    of linear equations formed by a fluxome.

    **See Also:**: Constant Field Values

---


### CONSTRAINT\_TOLERANCE

```
public static final java.lang.String CONSTRAINT_TOLERANCE
```

:   The keyword used to denote a constraint tolerance.

    **See Also:**: Constant Field Values

---


### DEFAULT\_CONSTRAINT\_TOLERANCE

```
public static final double DEFAULT_CONSTRAINT_TOLERANCE
```

:   The default constraint tolerance.

    **See Also:**: Constant Field Values

---


### requiredKeywords

```
public static final UnmodifiableSet<java.lang.String> requiredKeywords
```

:   The set of keywords required to be present in the `ManagerInput`
    objects supplied to instances of this class.

---


### optionalKeywords

```
public static final UnmodifiableSet<java.lang.String> optionalKeywords
```

:   The set of keywords understood, but not required, by instances of this
    class.


**Constructor Detail**

### FbaManager

```
public FbaManager()
```


**Method Detail**

### printFluxomeInfo

```
public static void printFluxomeInfo(Fluxome fluxome,
                                    java.io.PrintWriter out)
```

:   Prints basic information about the provided fluxome.

    :   **Parameters:**: `fluxome` - the fluxome for which basic information will be printed.: `out` - the print writer to which basic information will be printed.

---


### getRxnNameExprFormat

```
public static RxnNameExprFormat<LinearComb<java.lang.String>,java.lang.String> getRxnNameExprFormat(Fluxome f)
```

:   Returns the default reaction name expression format. This is currently
    `RxnNameExprFormatV1`.

    :   **Parameters:**: `f` - the fluxome for which the format will be created. **Returns:**: the default reaction name expression format.

---


### getConstraintTolerance

```
public static double getConstraintTolerance(ManagerInput<java.lang.String,java.lang.String> input,
                                            java.io.PrintWriter out)
```

:   Parses and returns the value for `CONSTRAINT_TOLERANCE` in the
    provided manager input.

    :   **Parameters:**: `input` - the input containing a value for `CONSTRAINT_TOLERANCE`.: `out` - the print writer to which a status message will be printed. **Returns:**: the parsed value for `CONSTRAINT_TOLERANCE` in the provided input. **Throws:**: `ApplicationException` - if the parsed tolerance is infinite, NaN, or < 0.

---


### getConstraintTolerance

```
public static double getConstraintTolerance(ManagerInput<java.lang.String,java.lang.String> input)
```

:   Parses and returns the value for `CONSTRAINT_TOLERANCE` in the
    provided manager input.

    :   **Parameters:**: `input` - the input containing a value for `CONSTRAINT_TOLERANCE`. **Returns:**: the parsed value for `CONSTRAINT_TOLERANCE` in the provided input. **Throws:**: `ApplicationException` - if the parsed tolerance is infinite, NaN, or < 0.

---


### getRxnNameHeaders

```
public static java.util.Set<java.lang.String> getRxnNameHeaders(ManagerInput<java.lang.String,java.lang.String> input,
                                                                Fluxome fluxome)
```

:   Parses and returns the value for `AbstractProcessManager.DATA_HEADERS` in the
    provided manager input. The format used to parse the data header
    string is `RxnNameSetFormatV1`.

    :   **Parameters:**: `input` - the input containing a value for `DATA_HEADERS`.: `fluxome` - the fluxome for which the data headers correspond. **Returns:**: the parsed value for `AbstractProcessManager.DATA_HEADERS` in the provided input.

---


### getRxnNameHeaders

```
public static java.util.LinkedHashSet<java.lang.String> getRxnNameHeaders(java.lang.String dataHeaders,
                                                                          Fluxome fluxome)
```

:   Parses and returns the set of reaction names contained in the provided
    string. The format used to parse the data header string is
    `RxnNameSetFormatV1`.

    :   **Parameters:**: `dataHeaders` - the data header string to be parsed.: `fluxome` - the fluxome for which the data headers correspond. **Returns:**: the set of reaction names contained in the provided string.

---


### getSingleFluxVectorOutputFile

```
public static MapOutputFile<java.lang.String,java.lang.Double> getSingleFluxVectorOutputFile(ManagerInput<java.lang.String,java.lang.String> input,
                                                                                             java.io.PrintWriter out,
                                                                                             Fluxome fluxome)
```

:   Returns an empty output file that is suitable for storing a single
    flux vector. The values for
    `FLUX_VECTOR_FILE_NAME`,
    `AbstractProcessManager.DATA_HEADERS`, and
    `AbstractProcessManager.FILE_FORMAT` in the provided input are parsed
    and used to call the method
    `getSingleFluxVectorOutputFile(String, FileFormat, Set, Fluxome)`.

    :   **Parameters:**: `input` - the input containing values for the listed keywords.: `out` - the print writer to which status messages will be printed.: `fluxome` - the fluxome for which the data headers correspond. **Returns:**: an empty output file that is suitable for storing a single flux vector. **See Also:**: `getRxnNameHeaders(ManagerInput, Fluxome)`, `AbstractProcessManager.getFileFormat(ManagerInput)`

---


### getSingleFluxVectorOutputFile

```
public static MapOutputFile<java.lang.String,java.lang.Double> getSingleFluxVectorOutputFile(java.lang.String fileName,
                                                                                             FileFormat fileFormat,
                                                                                             java.util.Set<java.lang.String> rxnNameHeaders,
                                                                                             Fluxome fluxome)
```

:   Returns an empty output file that is suitable for storing a single
    flux vector.

    :   **Parameters:**: `fileName` - the name of the file to be created.: `fileFormat` - the format of the file to be created.: `rxnNameHeaders` - the set of reaction names to be written to the file.: `fluxome` - the fluxome to which the reaction names correspond. **Returns:**: an empty output file that is suitable for storing a single flux vector.

---


### getMultiFluxVectorOutputFile

```
public static MapOutputFile<java.lang.String,java.lang.Double> getMultiFluxVectorOutputFile(ManagerInput<java.lang.String,java.lang.String> input,
                                                                                            java.io.PrintWriter out,
                                                                                            Fluxome fluxome)
```

:   Returns an empty output file that is suitable for storing multiple
    flux vectors. The values for
    `FLUX_VECTOR_FILE_NAME`,
    `AbstractProcessManager.DATA_HEADERS`, and
    `AbstractProcessManager.FILE_FORMAT` in the provided input are parsed
    and used to call the method
    `getMultiFluxVectorOutputFile(String, FileFormat, Set, Fluxome)`.

    :   **Parameters:**: `input` - the input containing values for the listed keywords.: `out` - the print writer to which status messages will be printed.: `fluxome` - the fluxome for which the data headers correspond. **Returns:**: an empty output file that is suitable for storing multiple flux vectors. **See Also:**: `getRxnNameHeaders(ManagerInput, Fluxome)`, `AbstractProcessManager.getFileFormat(ManagerInput)`

---


### getMultiFluxVectorOutputFile

```
public static MapOutputFile<java.lang.String,java.lang.Double> getMultiFluxVectorOutputFile(java.lang.String fileName,
                                                                                            FileFormat fileFormat,
                                                                                            java.util.Set<java.lang.String> rxnNameHeaders,
                                                                                            Fluxome fluxome)
```

:   Returns an empty output file that is suitable for storing multiple
    flux vectors.

    :   **Parameters:**: `fileName` - the name of the file to be created.: `fileFormat` - the format of the file to be created.: `rxnNameHeaders` - the set of reaction names to be written to the file.: `fluxome` - the fluxome to which the reaction names correspond. **Returns:**: an empty output file that is suitable for storing multiple flux vectors.

---


### getFluxome

```
public static IrrevRxnFluxome getFluxome(ManagerInput<java.lang.String,java.lang.String> input,
                                         java.io.PrintWriter out)
```

:   Opens the file denoted by `RXN_FILE` in the
    provided manager input and returns the fluxome it contains. The file
    denoted by `RXN_CATALYST_FILE` is also opened and applied if a
    value is provided.

    :   **Parameters:**: `input` - the input containing a value for `RXN_FILE` and `CATALYST_FILE`.: `out` - the print writer to which a status message will be printed. **Returns:**: the fluxome contained in the files indicated by `RXN_FILE` and `RXN_CATALYST_FILE` in the provided input.

---


### getFluxome

```
public static IrrevRxnFluxome getFluxome(java.lang.String fileName,
                                         java.io.PrintWriter out)
```

:   Returns the fluxome contained in the file with the provided name.

    :   **Parameters:**: `fileName` - the name of a file containing a fluxome parsable with `IrfFormatV1`.: `out` - the print writer to which a status message will be printed. **Returns:**: the fluxome contained in the file with the provided name.

---


### getFluxome

```
public static IrrevRxnFluxome getFluxome(java.lang.String fileName)
```

:   Returns the fluxome contained in the file with the provided name.

    :   **Parameters:**: `fileName` - the name of a file containing a fluxome parsable with `IrfFormatV1`. **Returns:**: the fluxome contained in the file with the provided name.

---


### getFluxome

```
public static IrrevRxnFluxome getFluxome(java.lang.String rxnFileName,
                                         java.lang.String catalystFileName)
```

:   Returns the fluxome contained in the files with the provided names.

    :   **Parameters:**: `rxnFileName` - the name of a file containing a fluxome parsable with `IrfFormatV1`.: `catalystFileName` - the name of a file containing reaction-catalyst associations parsable with BoolRelFormatV1 **Returns:**: the "catalyzed fluxome" contained in the files with the provided names.

---


### getFluxome

```
public static IrrevRxnFluxome getFluxome(java.lang.String rxnFileName,
                                         java.lang.String catalystFileName,
                                         java.io.PrintWriter out)
```

:   Returns the fluxome contained in the files with the provided names.

    :   **Parameters:**: `rxnFileName` - the name of a file containing a fluxome parsable with `IrfFormatV1`.: `catalystFileName` - the name of a file containing reaction-catalyst associations parsable with BoolRelFormatV1: `out` - the print writer to which descriptive message will be written. **Returns:**: the "catalyzed fluxome" contained in the files with the provided names.

---


### getCatalysts

```
public static java.util.Map<java.lang.String,Or<And<java.lang.String>>> getCatalysts(Fluxome fluxome,
                                                                                     java.lang.String fileName)
```

:   Returns the reaction-catalyst associations contained in
    the file with the provided name.

    :   **Parameters:**: `fluxome` - the fluxome with which reaction names are verified.: `fileName` - the name of the catalyst file. **Returns:**: reaction names mapped to the boolean logic statements governing their catalysis.

---


### getCatalysts

```
public static java.util.Map<java.lang.String,Or<And<java.lang.String>>> getCatalysts(Fluxome fluxome,
                                                                                     java.lang.String fileName,
                                                                                     java.io.PrintWriter out)
```

:   Returns the reaction-catalyst associations contained in the file with the
    provided name.

    :   **Parameters:**: `fluxome` - the fluxome with which reaction names are verified.: `fileName` - the name of the catalyst file.: `out` - the print writer to which descriptive messages will be written. **Returns:**: reaction names mapped to the boolean logic statements governing their catalysis.

---


### writeFluxome

```
public static void writeFluxome(IrrevRxnFluxome fluxome,
                                java.lang.String fileName)
```

:   Writes the provided fluxome to a file with the provided name. The
    output file format is `IrfFormatV1`.

    :   **Parameters:**: `fluxome` - the fluxome to be written to file.: `fileName` - the name of the file to which the fluxome will be written.

---


### writeFluxome

```
public static void writeFluxome(IrrevRxnFluxome fluxome,
                                java.lang.String fileName,
                                java.io.PrintWriter out)
```

:   Writes the provided fluxome to a file with the provided name. The
    output file format is `IrfFormatV1`.

    :   **Parameters:**: `fluxome` - the fluxome to be written to file.: `fileName` - the name of the file to which the fluxome will be written.: `out` - the print writer to which descriptive messages will be written.

---


### getFluxomeSolution

```
public static LinearSystemSolution<java.lang.String> getFluxomeSolution(ManagerInput<java.lang.String,java.lang.String> input,
                                                                        java.io.PrintWriter out,
                                                                        Fluxome fluxome)
```

:   Opens the file denoted by `SYSTEM_SOLUTION_FILE` in the
    provided manager input and returns the linear system solution
    it contains.

    :   **Parameters:**: `input` - the input containing a value for `FLUXOME_SOLUTION_FILE`.: `out` - the print writer to which a status message will be printed.: `fluxome` - the fluxome to which the solution corresponds. **Returns:**: the linear system solution contained in the file indicated by `SYSTEM_SOLUTION_FILE` in the provided input.


---


|  |  |  |
| --- | --- | --- |
| **Overview** \| **Package** \| **Class** \| **Use** \| **Tree** \| **Deprecated** \| **Index** \| **Help** | |  |
| **PREV CLASS**   **NEXT CLASS** | **FRAMES**    **NO FRAMES**     **All Classes** |  |
| SUMMARY: NESTED \| FIELD \| CONSTR \| METHOD | DETAIL: FIELD \| CONSTR \| METHOD |  |


---
